# Virtual Motivational Interviewing (VIMINT) to support physical activity: Experiences of older adults and counsellors

**DOI:** 10.1177/13591053241235094

**Published:** 2024-02-27

**Authors:** Olayinka Akinrolie, Jacquie Ripat, Shaelyn Strachan, Sandra C Webber, Ruth Barclay

**Affiliations:** University of Manitoba, Canada

**Keywords:** counsellors, motivational interviewing, older adults, virtual

## Abstract

The aim of this study was to explore the experiences of older adults and counsellors involved in virtual motivational interviewing (MI). This study was part of the Virtual Motivational INTerviewing (VIMINT) feasibility trial of virtual MI for physical activity in older adults. A descriptive qualitative design utilized content analysis. Seven older adults and three counsellors were interviewed. Four categories were developed: (i) Benefits and limitations of using technology (ii) Relationships between older adults and counsellors (iii) MI skills and spirit and (iv) Effects of virtual MI. Older adults and counsellors reported that receiving/delivering MI virtually was convenient and flexible. They described reduced non-verbal communication in virtual MI. Virtual MI facilitates interpersonal relationships, and counsellors reported that MI skills and spirit can be applied virtually. This study showed that virtual MI offers potential benefits with some limitations. The findings could inform future research involving virtual delivery of MI.

## Introduction

Motivational interviewing (MI) is a client-centred counselling style for resolving ambivalence and strengthening client commitment to change (Miller and Rollnick, 2013). MI emphasizes the importance of establishing good interpersonal relationships through a supportive environment (Rollnick et al., 2008). In MI practice, ambivalence is regarded as a normal process of change; when an individual is both ‘interested’ and ‘disinterested’ in changing behaviour (Miller and Rollnick, 2002). MI helps resolve ambivalence through a guiding style aimed at strengthening the intrinsic motivation for change through MI spirit; compassion, empathy, partnership and evocation (Rollnick et al., 2010). Compassion is an active process and involves giving priority to the individual’s needs (Miller and Rollnick, 2013). Empathy involves understanding the client’s perspective through a non-confrontational and non-judgemental approach with emphasis on the client’s autonomy (Miller and Rollnick, 2009). The process of collaboration between the counsellor and client is called partnership and evocation is used to elicit the client’s own motivation for change rather than instilling the counsellor’s views about change (Miller and Rollnick, 2013).

There are four processes in MI which include engaging, focusing, evoking and planning (Miller and Rollnick, 2002). Engaging involves establishing a therapeutic relationship with the client through building trust and using reflective listening. The process of maintaining direction towards change goals is called focus. While evoking is eliciting client’s own reason for change, it also remains an important process of MI. Planning refers to the client developing a change goal with support from the counsellor (Miller and Rollnick, 2013). Four basic skills are often used throughout MI. These include the use of open questions, affirmation, reflection and summary (OARS). These skills are essential for engaging, eliciting change talk and responding to sustain talk from client throughout the MI process (Miller and Rollnick, 2002). Change talk is an utterance that supports changing behaviour while sustain talk is an argument against change (Miller and Rollnick, 2013).

Originally developed in the field of addiction treatment, MI has been proven successful and applied to a wide range of behavioural health issues in adults and adolescents such as diabetes management (Ekong and Kavookjian, 2016), eating behaviours (Macdonald et al., 2012), smoking cessation (Lindson et al., 2019), weight loss (Barnes and Ivezaj, 2015) and behaviour change applied to other chronic conditions (Lundahl et al., 2010). A systematic review of randomized controlled trials by O’Halloran et al. (2014) found that MI had a modest effect on increasing physical activity levels among adults with chronic conditions. Although MI has been widely applied among adolescent and adult populations, researchers have suggested that the use of MI among older adults is limited (Akinrolie et al., 2020; O’Halloran et al., 2016). MI applied with the goal of improving health behaviours may be beneficial for older adults, aged 65 years and more (Cummings et al., 2009).

There is an increase in chronic disease prevalence with increasing age (Maresova et al., 2019). Over 60% of deaths in Canada are caused by four chronic diseases: cancer, cardiovascular disease, diabetes and chronic respiratory diseases (Statistics Canada, 2020). The onset of these diseases may be delayed and managed through behavioural health changes such as physical activity (Newsom et al., 2012). Counselling techniques such as MI have been shown to promote healthy behaviour and mitigate behavioural risk factors that are associated with chronic diseases (Lundahl et al., 2010).

Motivational interviewing (MI) is a complex technique that takes a considerable amount of time to learn and practice (Miller and Rollnick, 2009; Rollnick et al., 2008). According to Miller and Rollnick (2009), practicing MI without understanding and/or without exhibiting its underlying spirit is like using ‘words without a song’ (p. 131). The spirit of MI is the foundation of the MI counselling approach. Counsellors must have the required knowledge and skills to be able to deliver MI competently. Miller and Moyers (2006) highlighted stages in learning MI; for example, understanding the underlying spirit of MI, developing skills in client-centred OARS, eliciting change talk and developing a change plan. One of the instruments developed to assess counsellors’ competency and adherence is the MI Treatment Integrity scale (MITI) (Moyers et al., 2005). It has been shown to be a reliable scale for assessing both the relational and technical component of MI (Moyers et al., 2016). Groups such as the MI Network of Trainers were established to promote training and good practice by offering courses so as to ensure high quality delivery of MI (Motivational interviewing Network of Trainers, 2023). Greater counsellor competence and adherence to the MI principles has been associated with better outcomes (Lundahl et al., 2010; O’Halloran et al., 2014).

The use of technology (telehealth or telemedicine) for delivering interventions has rapidly increased due to the COVID-19 pandemic (Monaghesh and Hajizadeh, 2020; Wright and Caudill, 2020). Although the terms telehealth and telemedicine are often used interchangeably, the WHO (2021) defines telehealth as ‘the provision of healthcare services at a distance with communication conducted between healthcare providers seeking clinical guidance and support from other healthcare providers (provider-to-provider telemedicine); or conducted between remote healthcare users seeking health services and healthcare providers (client-to-provider telemedicine)’ (WHO, 2021: 2). Telehealth is associated with some advantages such as increased reach of healthcare, reduced traveling cost, reduced stress and convenience (Gajarawala and Pelkowski, 2021; Nittari et al., 2022). Several technologies have been deployed to deliver healthcare and counselling including telephone, email, internet, computer and videoconference (Balestra, 2018; Hailey et al., 2008). A combination of advances in technology and restrictions on movement imposed during COVID-19 contributed to the rapid use of real-time video and audio communication (Wright and Caudill, 2020).

Virtual technology is one form of video telehealth that can be integrated with various counselling methods, including MI. The term ‘virtual MI’ in this paper refers to the use of videoconferencing technology to transmit both video and audio of the counsellor-client interaction in real-time over the internet (Tzelepis et al., 2019). This excludes other forms of virtual means of delivering MI that have also been described, such as the use of animation, avatar and virtual worlds for delivering or training MI (Shingleton and Palfai, 2016; Woodruff et al., 2007). One of the advantages of videoconferencing over other forms of ‘virtual’ means is that it offers real-time face-to-face and voice-to-voice contact between the counsellor and participant (Wade et al., 2010). Compared with other technologies (e.g. telephone-delivered MI), virtual MI may be advantageous, since body language, and the spirit of MI can be expressed in real-time. Due to these advantages, virtual means of delivering MI could facilitate a greater rapport and collaboration between counsellor and participant compared to non-virtual MI (Kotera et al., 2021).

There are few studies that have explored the use of virtual MI for influencing health behaviours including physical activity (Alothman et al., 2022; Hawkins, 2010). A recent systematic review of the effectiveness of real time video counselling for smoking cessation found two studies with one using virtual MI (Tzelepis et al., 2019). This review reinforces the existing research gap in the use of virtual means to deliver MI. We are not aware of any studies that have used virtual MI in the older adult population. Understanding the experiences and interactions between older adults and counsellors in virtual MI may provide useful information about how MI skills and spirit can be applied in virtual format. Similarly, exploring the experiences of older adults could aid in understanding how they feel receiving MI through a virtual medium and its impact on their relationships with counsellors. Therefore, the purpose of this study was to explore the experiences of older adults and counsellors that participated in a virtual MI feasibility trial. Specifically, this study was designed to answer the following research questions:

(1) What are the experiences and perceptions of older adults receiving virtual MI to increase physical activity?
(2) What are the experiences and perceptions of counsellors providing virtual MI to older adults to increase physical activity?

## Methods

### Study design

This qualitative study was part of the Virtual Motivational INTerviewing (VIMINT) feasibility trial for older adults (trial registration no. NCT05179148) (Akinrolie et al., 2023). VIMINT is a single group pre- and post-design trial to examine the feasibility of using virtual MI to increase physical activity among older adults. Older adults received five sessions of virtual motivational counselling with sessions ranging from 10 to 60 minutes. The wide variation in the time of the sessions was due to the short duration of the last sessions. For example, the average duration of the first and second sessions was 46.57 ± 13.51 whereas the average duration of the fifth session was 32.85 ± 22.16. Each session was delivered by a counsellor who received training in MI. The VIMINT trial received ethics approval from the Health Research Ethics Board at the University of Manitoba (H2021:396).

A qualitative descriptive study design guided the data collection, analysis and reporting of this current study (Bradshaw et al., 2017). Qualitative description is a systematic way of describing, and exploring phenomena underpinning participants’ experiences (Elo and Kyngäs, 2008; Sandelowski, 2010). The aim of this design is to fully describe the phenomenon being studied and uncover perspectives, rather than providing evidence for existing ideas (Bradshaw et al., 2017). We followed the Consolidated criteria for reporting qualitative research (COREQ) (see Supplemental Material S1 for the checklist) (Tong et al., 2007).

### Participants

We interviewed both older adults and counsellors who took part in the VIMINT trial (Akinrolie et al., 2023). The older adults were eligible for the VIMINT trial if they met the following criteria: (1) aged 65 years or older, (2) able to walk with or without aids for at least 10 m, (3) living in the community, (4) reported that they engaged in less than 150 minutes of moderate-to-vigorous intensity physical activity per week, (5) scored ⩾18 on the telephone version of the Mini-mental State Exam (Roccaforte et al., 1992), (6) no medical health concerns reported in the Physical Activity Readiness Questionnaire Plus (PAR-Q+), or received clearance from their physician to participate (Warburton et al., 2014), (7) able to speak English, (8) had access to internet, and a computer or smart phone device. Older adults were recruited in Winnipeg through the local newspaper. Informed consent was obtained from eligible older adults, and they underwent assessment at baseline, post counselling and 2-month follow-up. All participants who completed the VIMINT trial agreed to take part in the qualitative study.

The counsellors were fourth year undergraduate students in kinesiology or psychology. All three counsellors involved in the VIMINT trial participated in the current study. One of the counsellors had completed a course in MI and the others had been involved in physical activity counselling using a client-centred approach. Each received 8 hours of training in MI including didactic and role-play. The training manual include the use of OARS skills and other skills used for eliciting change talk such evoking question, use of importance rule and querying extremes. The Motivational Interviewing Treatment Integrity Code (MITI 4.2.1) (Moyers et al., 2014) was used to assess competence in MI. The counsellors demonstrated good and fair competency in relational and technical components of MI respectively. Detailed information on the study procedures and training has been described elsewhere (Akinrolie et al., 2023).

### Data collection

Both older adults and counsellors were invited to participate in individual semi-structured interviews to explore their experiences and perceptions of VIMINT counselling. We developed separate interview guides for the older adults and for the counsellors. With the older adults, we asked open-ended questions to explore their experiences using technology, to discuss the virtual experience of receiving MI and to better understand their interactions with the counsellors. Counsellors were asked about their experiences interacting with older adult participants, the virtual experience of delivering MI and about the impact of the virtual format on developing relationships with the older adults. In addition, the counsellors were asked how the virtual format may have impacted the use of skills or spirit of MI. The semi-structured interviews were conducted on the videoconferencing platform (Zoom Video Communications Inc., 2016), and audio recorded. Permission was obtained before recording the interview. All the interviews were conducted by O.A, a male doctoral student who has experience conducting interviews and qualitative research (see Supplemental Material S2 and S3 for samples of interview guides used for older adults and counsellors). There was no relationship between the interviewer and participants.

### Analysis

The interviews were recorded and transcribed verbatim using Otter.ai (version 3.22.2, Otter.ai, Inc., Mountain View, CA). Author O.A then cleaned the transcript by listening to the audio and reviewing the transcripts for accuracy. A qualitative content analysis process was used, and data were analysed inductively (Bradshaw et al., 2017; Hsieh and Shannon, 2005). Our choice of content analysis was guided by our research questions. We aimed to systemically quantify and describe the experiences of the participants, and stay close to the data as much as possible (Vaismoradi et al., 2013). Data were managed manually using tables in Microsoft Word. The transcripts were first read independently by O.A, R.B. and J.R who familiarized themselves with the data and made sense of the data. To ensure trustworthiness and minimize subjectivity, two transcripts each from the older adults’ and counsellors’ interviews (four in total) were independently coded by the three authors. The unit of analysis was a phrase, or one or more sentences. The authors met, reviewed and discussed the codes, and developed an initial codebook. The codes were applied to the other transcripts by O.A. The new codes that emerged during the analysis of the remaining transcripts were added to the codebook. The analysts met again to review the application of initial codes and the development of new codes. Separate codebooks were developed for older adults and counsellors. Through analysis, we aimed to condense the broad description of the phenomenon (Bengtsson, 2016; Elo and Kyngäs, 2008), as described by the older adults and counsellors that were involved in the VIMINT study. Similar codes were grouped into sub-categories. Although developed sub-categories were from different groups of individuals, similar sub-categories from older adults and counsellors were reviewed by the analysts and merged to form categories after the older adult and counsellor transcripts were separately analysed (Carter et al., 2014; Farmer et al., 2006). Further analysis included comparing and contrasting the developed sub-categories and categories from the older adults’ interviews with those from the counsellors. The categories were triangulated with observation notes. Through an iterative process, we reflected on the developed categories in terms of their relevancy and meaningfulness to the research objectives.

## Results

**Figure 1.**
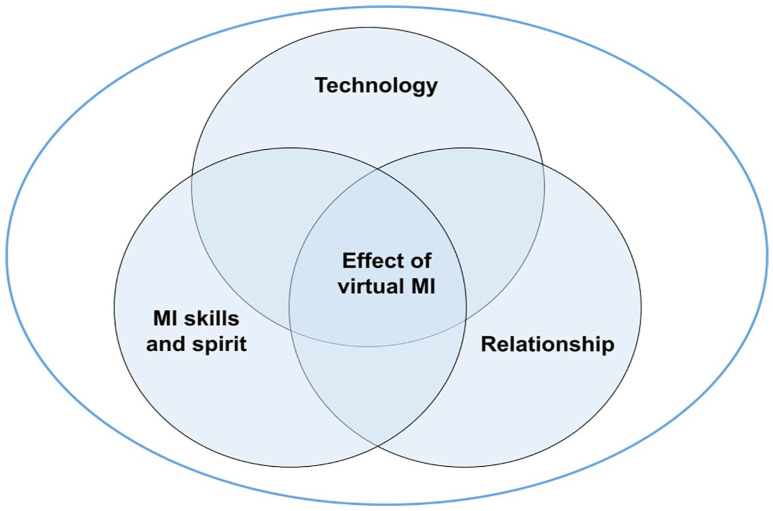
Virtual MI: conceptual relationship between technology, MI skills and spirit and relationship using virtual MI.

All the participants who completed the feasibility trial (*n* = 7) and all the counsellors (*n* = 3) participated in interviews. The average age of older adults was 68.9 ± 3.9 years (five females and two males). All the older adults were white, and six were retired. The counsellors included two females and one male. Four major categories were established from the experiences of older adults and counsellors involved in virtual MI: (i) *Benefits and limitations of using technology* (ii) *Relationship between older adults and counsellors* (iii) *MI skills and spirit* (iv) *Effects of virtual MI.* Categories III and IV were unique to counsellors and older adults respectively. Figure 1 illustrates the inter-relationship between the four categories in the context of virtual MI. Table 1 shows the codebook with examples of quotes from older adults and counsellors.

**Table 1.** Example of codebook showing the data analysis from participants and counsellors.

Examples of quote	Codes	Sub-categories	Categories
*Counsellors*			Benefits and limitations of using technology
*‘I guess overall, delivering the sessions over zoom was pretty easy. It felt pretty natural. I also, I mean, I have prior experience delivering interventions over zoom. So it’s not uncomfortable to me anymore’ (counsellor 1)*	Zoom experience was natural	Positive communication platform experience	
*‘. . .Even reading kind of like subtle facial expressions, or, like those kinds of nonverbal responses, and that type of thing, obviously, can’t quite be figured out in the same way as an in-person scenario’ (counsellor 2)*	Minimal non-verbal cues	Technological limitations	
*‘So whereas participants might feel like safer in their own environment. And there might be some more discomfort with coming into the laboratory, or into a, like, counseling room. . .’ (counsellor 1)*	Participants feel safer in virtual	Advantages of being in one’s environment	
*‘I think the good parts of it are, like I said, it’s more flexible, both for myself and for the participants. So I can take the meeting anywhere I am, they can take the meeting anywhere they are. . .’ (counsellor 1)*	Virtual was convenient and comfortable	Convenient and flexibility	
*‘But I found it a little more difficult to keep them on the call, they were very quick to want to end it and say, like, oh, well, if we’re done here’ (counsellor 3)*	Participants eager to end the call and leave the session	Virtual disadvantages	
*Participants*			
*‘I find zoom fine. I mean, I don’t do as much of it as some people do. So I don’t have that wealth of background but I find it certainly works as far as having a face to face communication and direct interaction’ (Participant 1, male, 65 years)*	Zoom was user friendly	Positive virtual experience	
*‘The fact is that, you know, we are inhibited by the necessity of looking at a screen, right, we have to focus on a screen. There’s no ambience here that we can actually benefit from. . .’ (Participant 2, female, 69 years)*	Screen limitation	Virtual limitation	
*‘And one thing that I had an issue with on my site was there was sometimes a weak connection’ (Participant 2, female, 69 years)*	Weak internet connection	Technological issue	
*‘Well, it just that it’s really easy. You don’t have to leave your house. You actually don’t even have to get out of your pajamas if you don’t want to. It’s really easy. I like it. . .’ (participant 3, female, 65 years)*	Virtual was convenient	Convenient factor	
*‘And the safety of myself because, you know, a lot of people don’t think that there’s COVID out there. Still COVID out there’ (participant 3, female, 65 years)*	Preference for virtual due to COVID-19 concern	Safety concerns	
*Counsellors*			Relationship between older adult and counsellor
*‘During the sessions themselves, I really enjoyed I thought I had a good connection with all the participants, and we had some good conversations’ (counsellor 1)*	Good connection with participants	Positive interaction with participant	
*I think it sort of comes back to that idea of we’re both in environments where we’re comfortable. And that’s, that can do a lot for building rapport. So if a participant is in a space where they feel safe and comfortable (counsellor 1)*	Virtual creates deeper connection with the participant	Impact of virtual on rapport and relationship with participant	
*Participants*			
*‘He really did and he remembered from session to session, something would be said in one session and he would bring it up again. You know, in another session, which was pretty impressive. So he was obviously listening to me’ (Participant 6, 77 female).*	Counsellor was a good listener and collaborator	Positive characteristic of counsellors	
*‘It was quite enjoyable, and the counsellor it’s very pleasant to talk with and very interested and interesting, which was kind of a nice change’ (participant 1, male, 65 years)*	Counsellor was good connection, affable and cordial relationship	Positive experience with counsellor	
*‘But the motivation provided really wasn’t very high. The interviewer was not a good motivator. Let’s put like that..’ (Participant 9, male, 72 years)*	Counsellor not good motivator	Undesirable characteristics of counsellor	
*Counsellors only* *‘I think in terms of ease to use was actually evoking change talk because the participants were within their own environments where they would have to be doing the physical activity. So they were able to look around in their own environments and think to themselves’ (counsellor 1)*	Virtual enables evoking	Virtual enhances MI skill	MI skills and spirit
*‘I think feeling empathy, expressing empathy through virtual MI. For me, it was probably the same as it would have been with in-person MI, I still felt empathy for the participants, I felt that I was able to express that empathy to them in a way that they, they perceived I was being empathetic’ (counsellor 2)*	Principle of MI not different in virtual context/easy to express empathy	Easy to adhere to MI principle	
*Participants only* *‘They did? Absolutely. But I which even though I had discussed this with Counsellor and said, we’ll start off with five or 10 minutes doing this this. I went a bit too “gung ho”’ (participant 8, female, 68 years).*	Increased PA	Improved PA and self-awareness	Effect of virtual MI
*‘Yes, (increased confidence), think I go between seven and eight (on a scale of 1–10)’ (participant 6, female, 77 years)*	Improved confidence and less anxiety	Improved self-efficacy	

### Benefits and limitations of using technology

This category captured the benefits and limitations of using virtual technology means of communication to deliver MI. Both older adults and counsellors spoke about advantages and limitations of virtual technology during MI. One of the advantages or positive experiences described by older adults was the use of the videoconferencing platform, Zoom. The majority of older adults said they did not have any problems navigating the platform, that it was familiar to them, and that it supported communication. Some reported that they had started using Zoom during the COVID-19 pandemic.

*I find Zoom fine. I mean, I don’t do as much of it as some people do. So I don’t have that wealth of background but I find it certainly works as far as having a face to face communication and direct interaction (older adult, male, 65 years)*

Using the videoconferencing platform was described by both older adults and counsellors as convenient and flexible. Counsellors expressed that MI was delivered naturally and effectively on Zoom. They reported that the convenience of doing the counselling session anywhere was comfortable as described below:

*Well, it is just that it’s really easy. You don’t have to leave your house. You don’t even have to get out of your pajamas if you don’t want to. It’s really easy. I liked it. . . (older adult, female, 65 years)*

*I think the good parts of it are, like I said, it’s more flexible, both for myself and for the participants [older adults] So, I can take the meeting anywhere I am, they can take the meeting anywhere they are. . . (counsellor 1).*

Some of the older adults appreciated the safety that virtual counselling offered. They expressed their preference for virtual counselling over in-person, as they would not have to be in contact with anybody while they were still concerned about COVID-19.

*And the safety of myself because, you know, a lot of people don’t think that there’s COVID out there. Still COVID (is) out there (older adult, female, 65 years)*

Also, counsellors narrated the advantages which virtual format could provide for older adults. They reported that this gave the older adults the ability to engage in their own familiar context. It enables the older adults to quickly and easily identified physical environmental barriers to physical activity and how the barriers could be overcome. In addition, older adults may feel safer in their own environment and the virtual format gave them more power than going to a lab or research environment.

*And so they will be at home, this is where they’re trying to get more active, and maybe taking that meeting at home, they’ll be a little bit more aware of the real-life barriers that have been coming up with them in terms of their physical activity (counsellor 1)*

Another advantage of using virtual technology described by the counsellors was that they were able to use the counselling guide during the MI session without participants being aware of it and thus not affecting the flow of the conversation.

*The nice thing about zoom actually is that I was able to have the file [the counselling guide] open on the side without the client knowing so then I can still kind of try to take a glance at it (counsellor 3)*

While participants expressed some advantages and positive experiences of virtual technology, they also expressed their concerns and limitations. Some of the limitations or disadvantages highlighted by the older adults were minimal non-verbal cues, lack of human contact, weak internet and security issues with technology. Most of the older adults reported that they could only see the counsellor’s face and shoulders during virtual counselling. Other parts of the body involved in non-verbal communication were not visible thereby affecting their interaction with the counsellor.

*. . .. there are a lot of nonverbal cues that can be picked up, of course, much better in a physical meeting, you know, than here [virtual], I mean, we’re dependent upon reading everything from our. . ., basically for the top of our head to the shoulders. . . (older adult 2, female, 69 years)*

The counsellors corroborated the older adults’ report on reduced non-verbal communication such as using hands and other body language in a virtual setting. This was expressed in the quote below:

*. . .Even reading kind of like subtle facial expressions, or, like those kinds of nonverbal responses, and that type of thing, obviously, can’t quite be figured out in the same way as an in-person scenario (counsellor 2)*

One counsellor expressed concerns about the ability to take turns talking due to the virtual nature of counselling. This was partly due to the inability to use non-verbal cues to signal older adults about their intention to talk.

*Like for the participant (older adults) I already mentioned, I had a lot of trouble being able to get a turn to speak. And I think a lot of that has to do with the fact that it was on Zoom (counsellor 2)*

Other limitations of technology reported by the counsellors included communication delays and occasional technical issues.

### Relationship between older adults and counsellors

Most of the older adults described positive experiences with the counsellors during virtual MI. They reported that the counsellors were good motivators, collaborators, affable and empathic. They believed that virtual MI was effective in developing rapport with counsellors, but the medium may not be optimal. They noted that the counsellors listened well and remembered what they had discussed from one session to the next. The conversation was described as a two-way dialogue, and they expressed their satisfaction with the session.

*Counsellor really did remember from session to session, something would be said in one session, and counsellor would bring it up again. You know, in another session, that was pretty impressive. So, counsellor was obviously listening to me (Older adult, female, 77 years)*

They felt that group virtual MI would provide an opportunity to connect not only with the counsellor but also with other people who share similar concerns. Others narrated challenges in developing a relationship with their counsellor, for instance when they were unable to communicate information effectively as described below:

*When I specifically asked for something and I don’t get a comment, the comment might be I’m sorry, that’s outside the boundaries of this study. But I didn’t really get an answer. It was just glossed over. . .. (Older adult, male, 72 years)*

All the counsellors reported that they had a positive connection, good rapport and were able to build trust with older adults. They believed that virtual format is a good medium to connect even though it has some limitations.

*So, I was happy about that. I find older adults to be a lot easier to talk to, they’re a lot more comfortable and confident in themselves. So, I think that is something that just makes the conversations flow pretty easily (counsellor 2)*

Counsellors also described how the virtual format impacted their connection with older adults. They felt that it could enhance openness and create a deep connection with the participant.

*I think it sort of comes back to that idea of we’re both in environments where we’re comfortable. And that can do a lot for building rapport. So, if a participant [older adult] is in a space where they feel safe and comfortable, then they might feel more willing to open up to their counsellor and talk to them about any struggles that they’re facing (counsellor 1)*

### MI skills and spirit

This category described the counsellors’ perspective as it relates to use of MI skills and adherent to MI counselling style. The counsellors reported that the MI skills represented by OARS, that is, Open-ended questions, Affirmation, Reflection and Summary, could successfully be applied virtually. In general, they reported the ability to use these MI skills easily in a virtual context.

*I think MI skills can definitely be applied virtually; I didn’t have any problems using any of the techniques. It’s all in the communication and communicating over Zoom was very easy (counsellor 1)*

However, one counsellor explained that it was somewhat difficult to use simple reflection (repeating what the older adult has said) due to communication delays experienced through the virtual platform. The counsellor stated that this affected verbal turn-taking between the older adult and counsellor during their discussion. Counsellors described how virtual mediums could facilitate processes of MI including engaging, evoking and planning. Counsellors suggested that adhering to the MI spirit- Partnership, Acceptance, Compassion and Evocation, are not influenced by virtual contexts. They felt that if a counsellor is genuinely empathic and passionate about helping others, providing virtual MI may not be different from in-person counselling.

*. . .like acceptance and compassion are not something that has to change for an online context, right? If you are here to try to legitimately help people and you care about them and want to listen to them, like you tried to be empathetic, so whatever barriers they might have or what their history might be. So, I think that wasn’t super hard to apply (counsellor 2)*

One of the counsellors reported difficulty adhering to some of the spirit of MI and the ability to obtain relevant information that could ultimately assist in developing plans. However, this counsellor noted that this challenge was not because of the virtual context but rather due to their own level of skill and competence.

### Effects of virtual MI

Virtual MI appeared to positively impact the older adults’ levels of physical activity, self-awareness and confidence in engaging in physical activity and overcoming barriers. Almost all participants reported an increase in physical activity and a plan to engage in physical activity after completing the virtual MI sessions. For instance, one older adult stated:

*I’m walking. . .. I’m going to try to walk three times a week at least for half an hour (Older adult, male, 72 years)*

Older adults described that they became more aware and conscious of their level of physical activity following the virtual MI with the counsellors. They reported becoming mindful of opportunities that would help improve their physical activity levels. One of the older adults described it as follows:

*I think I was more aware. And I said to myself, come on now you’ve got to move. You got to get out there and go for a walk in the morning when I woke up from the bed. I was thinking maybe I will go to the pool today. Oh no better go. . .. (Older adult, female, 65 years)*

Participants were asked about their confidence to engage in physical activity. Most of the older adults reported that they had become more confident and experienced less anxiety doing physical activity following the virtual MI sessions. The increased level of confidence they described was supported by the result from the confidence ruler (scale of 1–10, with ‘1’ and ‘10’ indicating ‘not confident at all’ and ‘completely confident’ respectively). Most older adults reported a high level of confidence as reported by one of the older adults:

*. . .Oh, 10 [level of confidence], yeah. Absolutely I have no lack of confidence. Thankfully, I will capitalize on the fact that I still have good health and I have still mobility (Older adult, female, 69 years)*

## Discussion

This study aimed to explore the experiences of older adults and counsellors involved in the VIMINT feasibility trial. Four categories were established: (i) Benefits and limitations of using technology, (ii) Relationships between older adults and counsellors, (iii) MI skills and spirit and (iv) Effects of virtual MI. Based on our findings, we conceptualized the inter-relationships between these categories in the context of virtual MI. We found that technology impacted communication negatively in terms of turn-talking and ability to recognize non-verbal cues. This may impact engagement and development of relationships between older adults and counsellors. In addition, this study showed that virtual technology had a positive impact on engagement between the older adults and counsellors. Also, our findings showed that technology could influence MI skills and spirit (see Figure 1).

Technology has potential benefits and challenges. One of our findings was that both older adults and counsellors felt that virtual MI was convenient and flexible. Across the literature, convenience has been identified as one of the main advantages of the virtual or online format of counselling (Baker and Ray, 2011; Mishna et al., 2015; Richards and Viganó, 2013). Virtual MI eliminates the need for older adults to travel to the counsellor’s office thereby saving time and money. It offers flexibility for both older adults and counsellors, allowing them to schedule sessions at any time of the day including outside of traditional office hours. One interesting finding in this study was that older adults expressed safety concerns due to COVID-19 infection and reported that the virtual delivery format could help prevent the spread of infection by eliminating human contact. This concern was appropriate as hospitalizations due to COVID-19 were still high in Canada at the time this study was conducted (between March and September 2022) (Government of Canada, 2022).

The disadvantages of technology for delivering MI highlighted by older adults and counsellors related to difficulties picking up non-verbal cues and lack of physical human contact. Previous studies on virtual counselling have also identified a limitation in recognizing non-verbal cues through the virtual medium or real-time video technology (Kotera et al., 2021; Otten et al., 2016). Recently, Kotera et al. (2021) explored the experiences of counsellors in the USA using a virtual format and practicing different types of behavioural counselling. Although they did not examine MI as a behavioural counselling method in their study, consistent with our study, they found that counsellors identified limited physical contact and body language as some of the challenges they faced during virtual counselling. The importance of non-verbal cues in communication and developing rapport has been established (Marcinowicz et al., 2010; Phutela, 2015). The lack of physical human contact as reported by older adults in this study seems to highlight the essence of face-to-face contact and how in-person interactions may facilitate better communication and relationships.

In terms of the relationships between older adults and counsellors in virtual MI, our findings showed that the virtual format delivery had a positive impact. In addition, counsellors felt that the virtual format could help build rapport and facilitate strong connections with older adults. This could be as a result of older adults being in their familiar environment. Khan et al. (2022) found that online formats, including videoconferencing, could be effective in developing therapeutic relationships between clients and counsellors. However, the counsellors in the study were practicing other types of counselling such as cognitive behavioural therapy, sex counselling and mindfulness (Khan et al., 2022).

Using MI skills and adhering to MI principles/spirit are fundamental to MI. Counsellors in our study reported that MI skills such as open questions and reflections were easy to apply. Researchers have questioned whether it is possible to express the underlying spirit of MI in other formats besides through in-person meetings (Shingleton and Palfai, 2016). Using automated computer prompts, emails, asynchronous videos and animated characters to deliver MI has been found to be problematic in showing appropriate empathy, partnership and developing interpersonal relationships with clients (Shingleton and Palfai, 2016). On the contrary, our study demonstrated that MI spirit can be adhered to and expressed in a virtual format that included synchronized real-time video and audio communication between counsellors and clients. Although limited, virtual MI may offer some levels of recognition of non-verbal cues compared to studies (e.g. Ang et al., 2013; Carels et al., 2007; Richards and Viganó, 2013) that used phone or text message communication.

Finally, this study showed that providing MI via virtual mean offers some benefits including increasing physical activity, self-efficacy and self-awareness. This is supported with the quantitative component of the earlier feasibility study (Akinrolie et al., 2023). In the feasibility study, we also observed changes in the physical activity level measured by accelerometry and self-reported measure. This finding is relevant because it shows that virtual means could be a promising way of providing MI and potentially effective in promoting physical activity among older adults. Although there are few studies that have used virtual MI for physical activity, our finding was consistent with that of Alothman et al. (2022) that found virtual MI increased physical activity among young adults.

### Study strengths and limitations

This study adds to the small number of studies that have explored the use of virtual MI and contributes findings from an older adult participant group. Also, exploring the experiences of counsellors expanded our understanding of delivering MI using a virtual means. However, there are some limitations that should be considered when interpreting the findings. Although the primary aim of qualitative research is not to generalize findings but rather provide an in-depth understanding of a phenomenon (Hays and McKibben, 2021; Leung, 2015), we were limited in the number of counsellors that we could recruit as only three counsellors were involved in the trial. Furthermore, the counsellors had limited MI experience, and none had previously delivered MI in-person, so they had to speculate about how virtual means affected applying MI skills and expressing MI spirit.

## Conclusion

This qualitative study provided useful findings about the experiences of older adults and counsellors using virtual MI. This study showed that there is an interconnection between technology, relationships and MI spirit that can result in effective MI delivery. Virtual MI has some potential benefits and limitations. One of the main benefits highlighted in this study was the convenience and flexibility it provided for both older adults and counsellors. One of the major drawbacks identified was the limited non-verbal cues that could be expressed virtually. Moreover, our study found that virtual MI could facilitate the development of interpersonal relationships as well as using MI skills and adhering to MI spirit. The findings of this study have implications for MI researchers. Virtual MI offers potential and promising benefits with some limitations. Increasing access through a virtual MI approach could be a valuable means of communicating with hard-to-reach individuals. In future studies, the perceptions of counsellors practicing MI virtually as well as in-person could be explored and compared. This will help us to understand how practicing and applying MI spirit with virtual means differs from in-person.

## Supplemental Material

sj-docx-1-hpq-10.1177_13591053241235094 – Supplemental material for Virtual Motivational Interviewing (VIMINT) to support physical activity: Experiences of older adults and counsellors Supplemental material, sj-docx-1-hpq-10.1177_13591053241235094 for Virtual Motivational Interviewing (VIMINT) to support physical activity: Experiences of older adults and counsellors by Olayinka Akinrolie, Jacquie Ripat, Shaelyn Strachan, Sandra C Webber and Ruth Barclay in Journal of Health Psychology
