# Supplementary material for: Virtual Motivational Interviewing (VIMINT) to support physical activity: Experiences of older adults and counsellors
Source: J Health Psychol. 2024 Feb 27;29(12):1416–30. doi: 10.1177/13591053241235094 (PMC11528923; doi:10.1177/13591053241235094)
Supplement: sj-docx-1-hpq-10.1177_13591053241235094 – Supplemental material for Virtual Motivational Interviewing (VIMINT) to support physical activity: Experiences of older adults and counsellors [file sj-docx-1-hpq-10.1177_13591053241235094.docx]

**Supplementary S1**

**Consolidated criteria for reporting qualitative studies (COREQ): 32-item checklist**

| **No. Item** | **Guide questions/description** | **Reported on Page #** |
| --- | --- | --- |
| **Domain 1: Research team and reﬂexivity** |  |  |
| *Personal Characteristics* |  |  |
| 1. Inter viewer/facilitator | Which author/s conducted the interview or focus group? | Page 8 |
| 2. Credentials | What were the researcher’s credentials? E.g. PhD, MD | Page 8 |
| 3. Occupation | What was their occupation at the time of the study? | Page 8 |
| 4. Gender | Was the researcher male or female? | Page 8 |
| 5. Experience and training | What experience or training did the researcher have? | Page 8 |
| *Relationship with participants* |  |  |
| 6. Relationship established | Was a relationship established prior to study commencement? | Page 8 |
| 7. Participant knowledge of the interviewer | What did the participants know about the researcher? e.g. personal goals, reasons for doing the research | NA |
| 8. Interviewer characteristics | What characteristics were reported about the interviewer/facilitator? e.g. Bias, assumptions, reasons and interests in the research topic | Page 8 |
| **Domain 2: study design** |  |  |
| *Theoretical framework* |  |  |
| 9. Methodological orientation and Theory | What methodological orientation was stated to underpin the study? e.g. grounded theory, discourse analysis, ethnography, phenomenology, content analysis | Page 8 |
| *Participant selection* |  |  |
| 10. Sampling | How were participants selected? e.g., purposive, convenience, consecutive, snowball | Page 7 |
| 11. Method of approach | How were participants approached? e.g., face-to-face, telephone, mail, email | Page 9 |
| 12. Sample size | How many participants were in the study? | Page 9 |
| 13. Non-participation | How many people refused to participate or dropped out? Reasons? | Page 9 |
| *Setting* |  |  |
| 14. Setting of data collection | Where was the data collected? e.g., home, clinic, workplace | Page 8 |
| 15. Presence of non-participants | Was anyone else present besides the participants and researchers? | Page 8 |
| 16. Description of sample | What are the important characteristics of the sample? e.g. demographic data, date | Page 9 |
| *Data collection* |  |  |
| 17. Interview guide | Were questions, prompts, guides provided by the authors? Was it pilot tested? | Page 8 |
| 18. Repeat interviews | Were repeat interviews carried out? If yes, how many? | NA |
| 19. Audio/visual recording | Did the research use audio or visual recording to collect the data? | Page 8 |
| 20. Field notes | Were ﬁeld notes made during and/or after the interview or focus group? | NA |
| 21. Duration | What was the duration of the interviews or focus group? | Page 9 |
| 22. Data saturation | Was data saturation discussed? | NA |
| 23. Transcripts returned | Were transcripts returned to participants for comment and/or correction? | NA |
| **Domain 3: analysis and ﬁndings** |  |  |
| *Data analysis* |  |  |
| 24. Number of data coders | How many data coders coded the data? | Page 8 |
| 25. Description of the coding tree | Did authors provide a description of the coding tree? | Page 9 |
| 26. Derivation of themes | Were themes identiﬁed in advance or derived from the data? | Page 9 |
| 27. Software | What software, if applicable, was used to manage the data? | Page 8 |
| 28. Participant checking | Did participants provide feedback on the ﬁndings? | NA |
| *Reporting* |  |  |
| 29. Quotations presented | Were participant quotations presented to illustrate the themes/ﬁndings? Was each quotation identiﬁed? e.g. participant number | Page 10-16 |
| 30. Data and ﬁndings consistent | Was there consistency between the data presented and the ﬁndings? | Page 16-20 |
| 31. Clarity of major themes | Were major themes clearly presented in the ﬁndings? | Page 10-16 |
| 32. Clarity of minor themes | Is there a description of diverse cases or discussion of minor themes? | Page 10-16 |

**Supplementary S2**

**Interview Guide for participant**

1. Can you tell me the reason why you joined this study?
2. What are your expectations before joining this study?

*Probe:*

*Were your expectations or needs met?*

*Was there anything that you experienced or happened during the counselling or the whole study that you did not expect?*

1. What was your experience doing the counselling on Zoom?

*Probe:*

*What are the issues you experienced using Zoom?*

*Is there anything you like about doing the MI counselling on Zoom?*

1. Do you think you could have had a different experience if the counselling was done in-person? How?
2. How can you describe your interaction with the counsellor?

*Probe:*

*Do you think it was easy to collaborate or partner with the counsellor via virtual means?*

*Does the counsellor listen to the issues you have raised and how was it addressed?*

*How well does the counsellor work with you throughout the sessions?*

*Does the counsellor see you as a partner or collaborator throughout the process?*

1. Did your confidence to continue physical activity changes following the MI session?

*Probe:*

*On a scale of 1 to 10 where 1 is ‘Not confident at all’ and 10 is ‘extremely confident’, How confident are you?*

1. What are your next steps regarding physical activity?

*Probe:*

*Have you made future physical activity goals?*

*Do you have a plan to achieve the goals?*

1. What do you think could have been done differently?

**Supplementary S3**

**Interview Guide for counsellors**

1. What are your experiences delivering motivational interviewing (MI) via the Zoom?

*Probe:*

*What are the challenges using virtual MI?*

*Is there anything interesting or good about using virtual MI?*

1. Do you think your experience could have been different if MI was delivered in-person?

*Probe:*

*What are your thoughts of using virtual MI compared to other means of delivering MI e.g., phone or in combination?*

*What are your thoughts about combining virtual MI and in-person MI?*

1. How can you describe your interaction with the participants using virtual MI?

*What impact do you think the virtual medium of delivering MI has on developing rapport and relationship with the participant?*

*Do you think your interaction would have been different if it was in-person?*

1. How can you describe the use of MI techniques and principles using virtual medium?

*Did you have any challenges using any of the techniques of MI using the virtual medium?*

*Do you think it was easy to adhere to the principles of MI using virtual medium?*

*What do you think about using the four principles of MI collaboration, compassion, acceptance and evocation via virtual mean?*

*Is there any stages of MI- engaging, evocation or planning that is easy to use well using virtual means?*

1. What are your thoughts on the number of sessions delivered to the participants?

Probe:

*Do you think the number of sessions was sufficient?*

*Do you think virtual mean has implication on the length of the session?*

*Do you think follow-up sessions could have helped?*

1. What do you think could be done to improve the delivery of virtual MI?
